# Microphysiological Systems for Comorbidity Studies: Chronic Kidney Disease and Osteoarthritis

**DOI:** 10.1002/adhm.202500550

**Published:** 2025-06-29

**Authors:** Mingying Han, Weiping Lin, Yuanxiong Cao, Patricia Murray, Bettina Wilm, Stephen J McWilliam, Jude Curran, Ruoxiao Xie

**Affiliations:** ^1^ Department of Materials Design and Manufacturing Engineering School of Engineering University of Liverpool Liverpool L69 UK; ^2^ Centre for Regenerative Medicine and Health Hong Kong Institute of Science and Innovation CAS Ltd. Fifth Affiliated Hospital of Guangzhou Medical University China; ^3^ Barts and the London School of Medicine and Dentistry Queen Mary University of London London E1 2AD UK; ^4^ Department of Physiology Anatomy and Genetics Kavli Institute for Nanoscience Discovery University of Oxford Oxford OX1 3QU UK; ^5^ Department of Women's and Children's Health Institute of Life Course and Medical Sciences University of Liverpool Liverpool L69 UK

**Keywords:** chronic kidney disease, comorbidity, multi‐organ‐on‐a‐chip, organ‐on‐a‐chip, osteoarthritis, microphysiological system

## Abstract

The global increase in chronic diseases and comorbidities due to aging populations is placing significant strain on healthcare systems. Chronic kidney disease (CKD) and osteoarthritis (OA) are among the most prevalent conditions in the elderly, with complex interconnections driven by shared risk factors such as systemic inflammation and metabolic dysregulation. Despite the critical need to understand these interactions, traditional animal models often fail to adequately capture the multidirectional crosstalk between human comorbid conditions, limiting insights into their mechanisms and complicating translational research. Advancements in microphysiological systems (MPS), which also known as organ‐on‐a‐chip (OoC) technologies, offer a promising alternative for studying comorbidities, such as CKD‐OA. MPS, which integrate human cells within biomimetic, bioengineered platforms, replicate the structural and functional properties of human tissues with unparalleled physiological relevance. This review explores the transformative potential of MPS technology in comorbidity research, focusing on CKD‐OA as a case study. Kidney‐on‐a‐chip and joint‐on‐a‐chip models and their applications in CKD and OA modeling are summarized and discussed. Furthermore, multi‐organ‐on‐a‐chip systems are = discussed for their potential to model comorbidities = and support the development of safer and more effective disease treatment strategies. This review underscores the potential of MPS to revolutionize comorbidity research and pave the way for personalized therapies.

## Graphical abstract

This review highlights the potential of organ‐on‐a‐chip systems for studying comorbidities, using chronic kidney disease (CKD) and osteoarthritis (OA) as examples. It summarizes recent advances in kidney‐on‐a‐chip and joint‐on‐a‐chip models and discusses their current and potential application in investigating CKD, OA, and CKD‐OA comorbidity, aiming to offer novel insights into future directions for engineered human disease models.

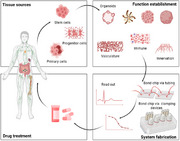

## 1 Introduction

As populations worldwide age, the prevalence of chronic diseases and comorbidities is continuously increasing, placing immense pressure on global healthcare systems.^[^ ^1^ ^]^ Chronic comorbidities, defined as the coexistence of two or more chronic diseases within one individual, often involve intricate mechanisms that complicate disease progression and treatment strategies. A deeper understanding of these interactions is essential for patients’ health.^[^ ^2^ ^]^

Chronic kidney disease (CKD) and osteoarthritis (OA) are among the most prevalent chronic conditions in the elderly population globally.^[^ ^3^, ^4^ ^]^ CKD, characterized by the progressive loss of kidney function, impacts more than 10% of the general population globally, amounting to over 800 million individuals. Among those aged over 65 years, the prevalence of CKD increases to ≈44%.^[^ ^5^ ^]^ OA, a degenerative joint disease, affected ≈595 million people globally in 2020, accounting for ≈7.6% of the world's population.^[^ ^6^ ^]^ The prevalence of OA increases with age: ≈38.4% of individuals aged 70 and older, 23.2% in the 50–69 age group, and ≈3.0% of younger adults aged 25–49 are affected by OA.^[^ ^7^ ^]^ The age‐related prevalence of these diseases underscores the importance of further research as the global population continues to age. In addition to aging, CKD and OA also share common risk factors, such as systemic inflammation and metabolic dysregulation. Some comorbidities common in OA patients, such as obesity and hypertension, are also known to increase the risk of CKD.^[^ ^8^, ^9^, ^10^, ^11^ ^]^ These factors lead to an increased prevalence of CKD in patients with OA than in those without.^[^ ^4^, ^12^ ^]^ The dialysis‐dependent CKD population represents a high‐risk group for OA partially due to the accumulation of uremic toxins which promote chondrocyte oxidative stress and upregulate matrix metalloproteinases (MMPs), driving matrix degradation.^[^ ^13^ ^]^ Also, the mineral bone disorder that often happens with CKD induces subchondral bone sclerosis and altered biomechanics, both of which exacerbate cartilage breakdown. Meanwhile, OA patients show a higher prevalence of CKD, potentially related to the chronic use of NSAIDs, aging, and comorbidities common which are considered as high risk factors for CKD.^[^ ^14^ ^]^ According to data from the United States National Health and Nutrition Examination Survey (2011–2020), 26.69% of OA patients had concurrent CKD, compared to only 13.83% among non‐OA individuals.^[^ ^15^ ^]^ Although it is recognized that the coexistence of these chronic diseases can complicate treatment strategies and clinical outcomes, research into their mechanisms and combined impact remains limited.

Traditional preclinical animal models typically focus on specific single disease, overlooking the bidirectional/multidirectional interactions of comorbid conditions.^[^ ^16^, ^17^, ^18^, ^19^, ^20^, ^21^ ^]^ Comorbidities may arise from distinct mechanisms but interact dynamically as they progress, creating feedback loops that complicate disease management.^[^ ^22^ ^]^ Recognizing this gap, researchers have begun developing models to study comorbid conditions. For instance, Christopher et al. developed the first preclinical comorbid CKD‐OA model using mice.^[^ ^23^ ^]^ They discovered many bidirectional disease‐modifying interactions between CKD and OA, which support the importance of studying CKD‐OA crosstalk. For example, they demonstrated that CKD alone (5/6 nephrectomy) drove early OA‐like bone changes, including increased synovial MMP‐13 expression and subchondral osteoclast activity, while OA alone (meniscal destabilization) induced renal fibrosis and proteinuria. Interestingly, when meniscal destabilization was performed on a CKD background, it significantly attenuated cartilage damage and osteophyte formation compared with OA alone mice. Although the emerging animal‐based models provide valuable insights, they face inherent limitations due to physiological and genetic differences between humans and animals. These differences can obscure disease mechanisms and hinder the translation of findings to human contexts. Moreover, animal studies are often constrained by ethical concerns, high costs, and the time‐intensive nature of in vivo experiments, highlighting the need for alternative approaches.

In recent years, advances in stem cell technologies, microfluidics, and biofabrication have led to the development of microphysiological systems (MPS), also known as organ‐on‐a‐chip (OoC) technologies. OoCs are bioengineered platforms designed to replicate the functional and structural properties of human tissues and organs in vitro. The biomimetic architecture and function of OoCs are usually achieved by combining 3D bioengineered constructs (e.g., cell‐laden hydrogels,^[^ ^24^ ^]^ differentiated stem cells,^[^ ^25^ ^]^ multicellular spheroids,^[^ ^26^ ^]^ and organoids,^[^ ^27^ ^]^) ex vivo tissues (e.g., biopsies, explants),^[^ ^28^, ^29^, ^30^ ^]^ re‐cellularized scaffolds,^[^ ^31^ ^]^ or bio‐printed constructs with microfabricated structures,^[^ ^32^, ^33^ ^]^ and possibly active stimulations (electrical, biochemical, or mechanical stimuli).^[^ ^34^, ^35^, ^36^ ^]^ OoC technology is transformative because it addresses many of the limitations inherent in traditional preclinical models. By integrating human cells, OoCs enable the study of human‐relevant disease mechanisms that are difficult to capture in animal models. Offering unparalleled opportunities for mimicking the complex microenvironment of human tissues and organs in vitro, OoCs also provide a more ethical alternative to traditional animal testing.^[^ ^37^ ^]^ Unlike conventional two‐dimensional cell culture systems, which fail to replicate the three‐dimensional architecture and microenvironment of native tissues, OoCs provide a more biomimetic setting where cells exhibit realistic morphology, behavior, and responses to stimuli. For example, perfused microfluidic channels in these systems can replicate blood flow, nutrient delivery, and waste removal in vascularized tissues. The inclusion of mechanical cues, such as cyclic strain or fluid shear stress, mimics the physical forces experienced by tissues in vivo, enhancing the physiological relevance of these models.^[^ ^38^, ^39^ ^]^ Incorporating these biomimetic features into OoC models is essential for recapitulating CKD and OA disease phenotypes in vitro. CKD is typically characterized by glomerular damage (e.g., sclerosis and hyalinosis), tubulointerstitial fibrosis with tubular atrophy, arteriosclerosis of renal vessels, resultant proteinuria, and disordered mineral metabolism (e.g., calcium/phosphorus imbalance). Microfluidic technology excels at mimicking kidneys by incorporating complex tubular architectures, dynamic fluidic environments, and the capacity for permeability assays. Through controlled shear stress and the introduction of injury‐inducing factors (e.g., nephrotoxic drugs), kidney‐on‐a‐chip models can be used to study impaired renal functions. OA is a degenerative joint disease involving synovial inflammation, cartilage breakdown, subchondral bone changes, and osteophyte formation. OoCs are able to replicate the osteochondral unit (cartilage, bone, and synovium) and apply mechanical loading to mimic joint hyperphysiological stress, reflecting disease conditions. Crucially, OoCs provide the capability to integrate multiple organ systems and disease models within a single platform, enabling the study of complex inter‐organ interactions and comorbidities,^[^ ^40^ ^]^ Thus, they would allow for the investigation of how CKD‐related inflammation and metabolic waste influence OA progression and how OA‐derived mediators affect kidney function, which traditional models struggle to capture due to species differences or oversimplification. These systems also facilitate real‐time observation of disease progression and therapeutic responses, offering unprecedented insights into bidirectional and multidirectional crosstalk.^[^ ^41^ ^]^ Given their transformative potential, OoCs represent a promising tool for advancing our understanding of complex comorbid conditions, like CKD‐OA comorbidity.

**Figure 1:**
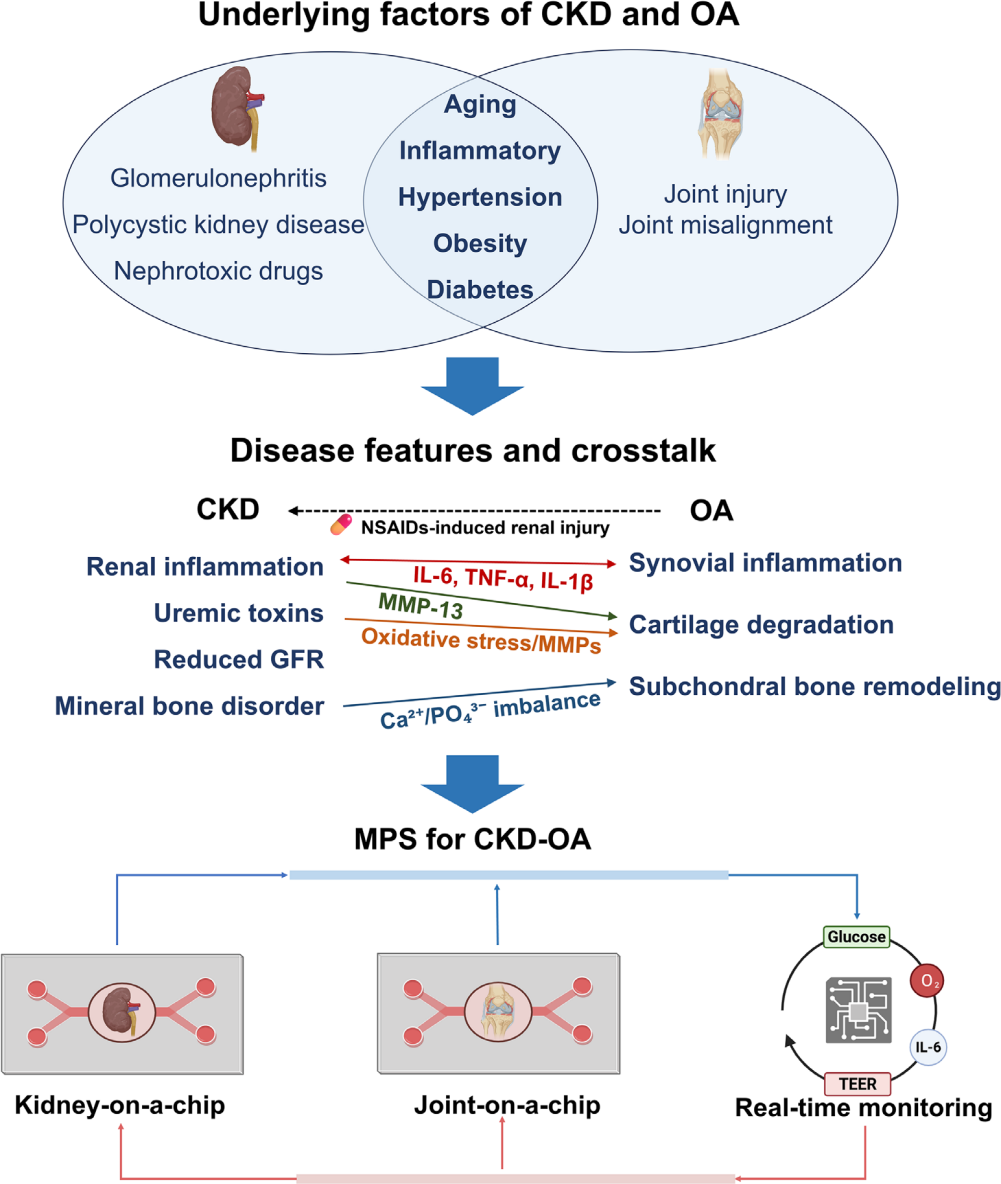
MPS for understanding the complications of CKD and OA. Figure created with BioRender.com. Abbreviation: GFR, glomerular filtration rate.

In this review, we delve into the transformative potential of OoC technology in advancing comorbidity research, with a particular emphasis on CKD‐OA comorbidity, a growing concern due to the increasing prevalence of both diseases worldwide (**Figure** **1**). We begin by providing an overview of existing kidney‐on‐a‐chip and joint‐on‐a‐chip models and examining their applications in replicating the pathophysiology of CKD and OA. Furthermore, we discuss the need and challenges in developing human‐relevant in vitro models that capture the interplay between organs and how these developments can be used for enhancing our understanding of disease mechanisms and improving personalized treatment strategies for comorbidities, like CKD‐OA comorbidity. These discussions highlight the potential of OoCs to revolutionize comorbidity research and pave the way for more effective healthcare strategies.

## 2 Kidney Microphysiological Systems and Chronic Kidney Disease

### 2.1 Renal Physiology and Chronic Kidney Disease

The kidney plays a crucial role in filtering waste products, toxins, and excess fluid from the blood, maintaining electrolyte balance, as well as regulating blood pressure and systemic homeostasis.^[^ ^42^ ^]^ The nephron is the structural and functional unit of the kidney, which is located across the cortex and the medulla.^[^ ^43^ ^]^ Each nephron contains a renal corpuscle connected to a renal tubule surrounded by peritubular capillaries.^[^ ^44^ ^]^ The renal corpuscle is primarily composed of the glomerulus\ surrounded by the Bowman's capsule. In the glomerulus, blood is filtered via a specialized glomerular basement membrane (GBM) surrounded by a complex structure of glomerular endothelial cells, podocytes, and mesangial cells.^[^ ^45^ ^]^ In general, molecules smaller than 2 nm can cross the GBM while all molecules larger than 4.2 nm cannot. The ability of molecules between 2 and 4.2 nm to cross the GBM depends on their charge and shape. The renal tubule is composed of a spatial alignment of epithelial cells and can be divided into three sections: proximal tubule, the intermediate tubule, and the distal tubule. The specific function of the renal tubule is to reabsorb water and other useful substances toward the circulatory system.^[^ ^46^ ^]^ The proximal tubule is involved in active solute secretion, hormone production, and various metabolic functions of the kidney.^[^ ^47^ ^]^ Apart from these key functional cells, stromal tissues and vasculature also contribute to the function and complexity of the kidney.

**Figure 2:**
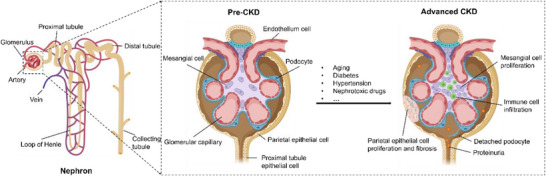
Nephron structure and cellular CKD progression. Figure created with BioRender.com.

CKD refers to the gradual loss of kidney function over time (**Figure** **2**). The defining pathological feature of CKD is the gradual and irreversible loss of functional nephrons, accompanied by a progressive decline in the glomerular filtration rate (GFR). This process is believed to result from persistent or recurrent damage to the kidney, ultimately causing fibrosis and scarring of the organ.^[^ ^48^ ^]^ The causes of CKD are varied, such as chronic glomerulonephritis, high blood pressure, diabetes, nephrotoxic drugs, aging, and hereditary nephropathy. The sustained injury results in the loss of nephrons and can induce inflammation, which further promotes nephron damage.^[^ ^49^ ^]^ When CKD progresses to end‐stage kidney failure, dialysis or kidney transplantation is required to sustain life. Effective preclinical models are thus urgently needed to improve our understanding of CKD development and facilitate the development of effective treatments.

### 2.2 Kidney Microphysiological Systems

Traditional 2D cell cultures lack key phenotypes, such as segment‐specific tubular morphology, apical‐basal polarity, and the presence of physiologically relevant cell‐cell and cell‐matrix interactions, and functions, such as selective reabsorption and secretion, flow‐induced mechanotransduction, and the maintenance of glomerular filtration and tubular barrier integrity.^[^ ^50^ ^]^ Animal models, however, suffer from the genetic differences between animals and humans.^[^ ^51^ ^]^ The OoC technology holds promise for a more physiologically relevant kidney model. Kidney MPS (kidney‐on‐a‐chip models) are platforms with the distinctive capacity to mimic the physiological structural, microenvironments of the human kidney and multiorgan coculture which makes kidney‐on‐a‐chip competitive in modeling disease in vitro.^[^ ^52^ ^]^ As the kidney and even its functional unit, the nephron, possess complex structures and functions, researchers are facing great difficulties in recapitulating their complete functions in vitro. The nephron is a highly polarized, tubular system with a distinct apical–basolateral axis critical for directional transport. There are over 20 specialized renal cell types (e.g., podocytes, glomerular endothelial cells, mesangial cells, proximal tubule epithelial cells, loop of Henle cells) with highly distinct phenotypes, tightly organized spatial arrangements, and functionally specialized roles.^[^ ^53^ ^]^ The functionality of nephron units relies on the precise interactions between different cell types and segments. However, replicating this complexity in vitro remains a significant challenge due to the need to integrate multiple cell types, recreate structural topography, replicate specialized extracellular matrix (ECM) environment, and incorporate interactions with the immune system. Current studies have to settle for less by focusing on a few key functions of the nephron, such as developing a glomerular model to investigate glomerular filtration or a renal tubule model to study reabsorption, nephrotoxicity, and disease effects in tubule segments, depending on the research question. In the following sections, we discuss the major developments and challenges of kidney‐on‐a‐chip in the construction of glomeruli and renal tubules and their potential to replicate CKD. We also summarized cell types, fabrication techniques, matrix, and functional assay used in current kidney‐on‐a‐chip models as well as their advantages and limitations in **Table** **1**.

**Table 1:** Summary of kidney‐on‐a‐chip models.

Model	Cell types	Fabrication	Matrix	Advantages	Limitations	Functional assay	Refs.
Glomerulus‐on‐a‐chip	iPSC‐derived podocytes, human glomerular endothelial cells	Stereolithography for microfluidic with stretchable walls, patterned silicon wafer for porous PDMS membrane	Porous PDMS membrane with laminin coating	Flow, stretchable, barrier formation	Limited 3D capillary geometry, lack of immune components	Inulin ‐FITC, albumin‐ Alexa Fluor 555	[58]
	Human primary podocytes and glomerular endothelial cells	Compression molding, ECM lane	Collagen I	Flow, high throughput, ECM interaction, barrier formation, permselectivity	Limited precise architectural control, lack of immune components	Albumin‐FITC, inulin‐FITC, PAN exposure	[60]
	E11 murine podocytes, primary vein endothelial cells	Microfluidic spinning	RGD‐conjugated alginate	Complex concave and convex topographies over multiple length scales, high throughput	Alginate limited in long‐term culture, lack of immune components	Albumin ‐FITC, doxorubicin exposure	[61]
	Human umbilical vein endothelial cells	Multiphoton guided bioprinting	Collagen I	High resolution, cellularized glomerular structure	Requires advanced equipment, low efficiency, lack of podocytes, lack of permeability assay	Red blood cell flow	[62]
Proximal tubule‐on‐a‐chip	RPTECs, renal microvascular endothelial cells	Photolithography for microfluidic	Porous PDMS membrane with collagen IV and Matrigel coating	Flow, barrier formation	Porous membrane limited cell crosstalk, low efficiency, lack of immune or interstitial components	Transporter function: Digoxin, Metformin, TEA, PAH	[75]
	RPTECs	Compression molding, ECM lane	Collagen I	Flow, high throughput, ECM interaction, barrier formation	Limited precise architectural control, lack of endothelial cells, lack of immune or interstitial components	TRITC‐dextran (4.4 kDa), FITC‐dextran (155 kDa), cisplatin exposure	[78]
	RPTEC/TERT1, glomerular microvascular endothelial cells	Placing two wires as templates in multiplexed chip device	Gelatin–fibrin gel	Flow, using wire templating to realize high throughput, ECM interaction, barrier formation,	Lack of complicated structures, lack of immune or interstitial components	RNA‐seq	[79]
	OPTECs	Fishing line (100–380 µm in diameter) as channel templates in multiplexed chip device	Gelatin–fibrin gel	Flow, 3D tubular structures, barrier formation	Lack of complicated structures, lack of immune or interstitial components	Inulin‐FITC, cisplatin or aristolochic acid exposure,	[84]
	RPTEC/TERT1, glomerular microvascular endothelial cells	Extrusion‐based 3D printing	Gelatin–fibrin gel	Flow, barrier formation, reabsorption	Low efficiency, lack of immune or interstitial components	Inulin‐FITC, dextran‐FITC, Albumin and glucose reabsorption	[86]
Kidney organoid‐on‐a‐chip	Kidney organoids derived from iPSCs	Multimaterial 3D bioprinter for chips fabrication	Gelatin–fibrin gel	Flow, vascular network formation	Immature, lack of perfusion into the nephrons and capacity for functional tests lack of immune or interstitial components	Doxorubicin exposure	[89]
	Kidney organoids derived from ESCs, peripheral blood mononuclear cells	Multimaterial 3D bioprinter for chips fabrication	Gelatin–fibrin gel	Flow, immune cell integration.	Immature, lack of perfusion into the nephrons, and capacity for functional tests	Assessing T cell bispecific antibodies	[90]

#### 2.2.1 Glomerulus‐On‐A‐Chip Models

**Figure 3:**
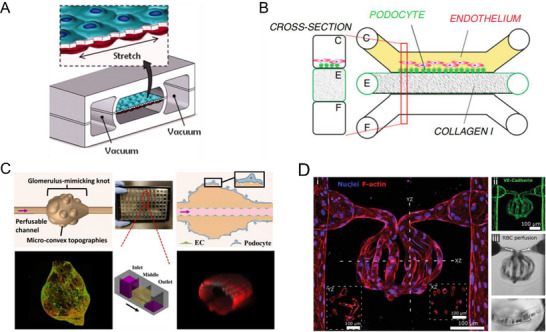
Glomerulus‐on‐a‐chip models. A) The microfluidic device features compartmentalized PDMS microchannels separated by a thin, porous, and flexible PDMS membrane coated with ECM. A capillary barrier is formed across this membrane, and physiological mechanical motions are simulated by applying cyclic vacuum to adjacent side chambers, inducing controlled stretching of the membrane. Reproduced with permission.^[^ ^57^ ^]^ Copyright 2010, American Association for the Advancement of Science. B) A high‐throughput glomerulus‐on‐a‐chip model was developed by co‐culturing podocytes and endothelial cells on collagen I‐based tubular structures, successfully recapitulating the glomerular filtration barrier, as demonstrated by a permselectivity assay using FITC‐labeled albumin. Reproduced (Adapted) under the terms of the CC‐BY license.^[^ ^60^ ^]^ Copyright 2019, Springer Nature. C) The device integrates a microfluidical extruded topographic hollow fiber composed of a perfusable vessel‐like tubular channel for endothelial cell culture and a glomerulus‐like knot with microconvex topography for podocyte attachment. Assembled in a 96‐well plate format, this platform enables scalable fabrication and high‐content screening. Reproduced (Adapted) under the terms of the CC‐BY license.^[^ ^61^ ^]^ Copyright 2022, American Chemical Society. D) Multiphoton‐guided fabrication enables the construction of a cellularized glomerulus‐on‐a‐chip with functional vascular perfusion. (i) Immunofluorescence imaging shows a 3D reconstruction of the glomerular structure with orthogonal cross‐sections (YZ and XZ planes). (ii) VE‐cadherin staining highlights the integrity of endothelial junctions. (iii) Functional perfusion is demonstrated by red blood cell (RBC) flow, with magnified views capturing single‐cell transit within the microvascular network. Reproduced (Adapted) under the terms of the CC‐BY license.^[^ ^62^ ^]^ Copyright 2021, Wiley.

The kidney glomerulus consists of specialized capillaries, where endothelial cells form the inner layer of the capillary wall on the vascular side, while podocytes cover the outer surface on the ultrafiltrate side.^[^ ^54^ ^]^ As the glomerulus is the primary filtering unit of the kidney, most kidney diseases manifest initially as dysfunction within this structure.^[^ ^55^ ^]^ Estimates from experimental and computational studies suggest that the shear stress in glomerular capillaries typically ranges from 1 to 5 dyn cm^−^^2^.^[^ ^56^ ^]^ Accurately mimicking this shear stress in vitro is crucial for developing physiologically relevant glomerulus‐on‐chip models. In addition, the permeability assay is essential for assessing the glomerular filtration barrier, which should allow small molecules (e.g., inulin) to pass while restricting large proteins (e.g., albumin). This selective filtration can be confirmed in in vitro models by using labeled albumin, dextran, or other size‐specific tracers. The microfluidic device, with a porous and stretchable polydimethylsiloxane (PDMS) membrane sandwiched between perfusable channels, has been created for replicating various biomimetic barriers. (**Figure** **3**A).^[^ ^57^ ^]^ Musah et al. utilized this design to co‐culture induced pluripotent stem cells (iPSCs) derived podocytes and endothelial cells, which have been shown to promote the maturation of the podocytes and reconstruct the selective permeability, as evidenced by permeability assay.^[^ ^58^ ^]^ Besides, the system imposed various flow speeds to realize a shear stress of 0.0007 dyn cm^−2^ for the urinary channel and 0.017 dyn cm^−2^ for the capillary channel, which effectively recapitulates the distinct hemodynamic and urinary flow conditions present in the native glomerulus. However, this system still has limitations, as the PDMS porous membrane poorly mimics the GBM between endothelial and podocyte layers and could potentially affect the proper crosstalk between cells. Furthermore, PDMS is prone to non‐specific adsorption of proteins, cytokines, and small molecules,^[^ ^59^ ^]^ which will also compromise the physiopathological relevance of the model. Attempts have also been made to develop glomerular chips that replicate the glomerular filtration barrier by co‐culturing endothelial cells and podocytes on collagen I scaffolds within high throughput platforms (Figure 3B).^[^ ^60^ ^]^ The gravity‐driven perfusion system enabled a dynamic flow that generates the shear stress. Further advancements in the microfabrication of biomaterials have enabled novel strategies to mimic the intricate architecture of the glomerulus. For example, Xie et al. innovatively utilized microfluidic spinning to recapitulate the tubular structure as well as complex concave and convex topographies of the glomerulus over multiple length scales (Figure 3C).^[^ ^61^ ^]^ This microfluidic spinning strategy allows the rapid fabrication of meter‐long topographic hollow hydrogel fiber within minutes. The resulting hydrogel fiber is composed of a vessel‐mimicking perfusable tubular channel and glomerulus‐like knots with surface topographies that support podocytes’ maturation. Other attempts have also been made to improve the recapitulation of the glomerular structures. For example, Rayner et al. successfully created 3D perfusable microchannels mimicking the glomerulus using a multiphoton ablation technique, which offers both high resolution and precise 3D control.^[^ ^62^ ^]^ These microchannels were cellularized with endothelial cells, perfused, and exhibited significant geometric complexity at capillary scale (Figure 3D).

#### 2.2.2 Renal Tubule‐On‐A‐Chip Models

Given the renal tubule's essential roles in solute reabsorption, secretion, and homeostatic regulation, and the high prevalence of renal disorders such as CKD, there is growing interest in modeling the renal tubule.^[^ ^63^ ^]^ The brush border formed on the apical membrane of the renal tubule is extremely crucial for the reabsorption of glomerular filtrate.^[^ ^64^ ^]^ The calculated apical fluid shear stress acting on the epithelial cells lining the renal tubule is ≈0.1 to 1 dyne cm^−2^.^[^ ^65^ ^]^ A transwell system is a conventional method applied to study renal proximal tubular function and pathology.^[^ ^66^ ^]^ However, this method is limited due to the lack of fluid shear stress, which is essential for the maturation and functions of the proximal tubule epithelial cells (PTECs).

**Figure 4:**
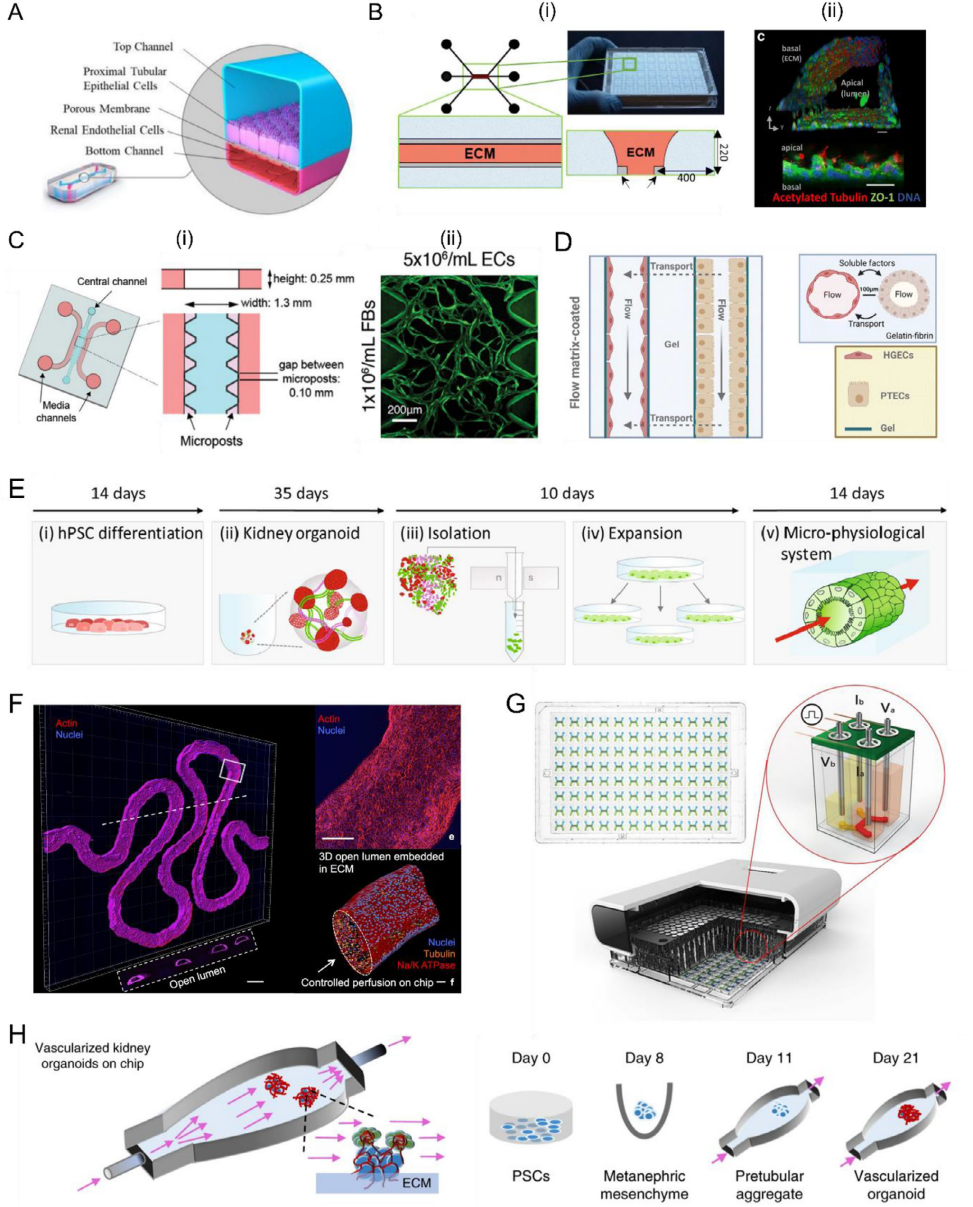
Renal tubule‐on‐a‐chip and kidney organoid‐on‐a‐chip models. A) Schematic illustration of the renal proximal tubule‐on‐a‐chip. RPTECs are cultured in the upper channel, while renal microvascular endothelial cells are cultured in the lower channel, separated by a porous membrane coated with ECM. Reproduced under the terms of the CC‐BY‐ND license.^[^ ^75^ ^]^ Copyright 2022, Anantha Ram Nookala. B) (i) Schematic illustration of Nephroscreen, including two perfusable channels and ECM separation. (ii) The image of the tubule showed the presence of cilia (acetylated tubulin, red) pointing in the direction of the lumen. The confluent tubules exhibited ZO‐1 expression (green) at cell borders, confirming the formation of tight junctions between adjacent epithelial cells. Reproduced (Adapted) under terms of the CC‐BY license.^[^ ^78^ ^]^ Copyright 2018, Springer Nature. C) (i) Schematic illustration of microfluidic with microposts for microvasculature formation and (ii) confocal image of endothelial cells and fibroblasts. Reproduced (Adapted) under the terms of the CC‐BY license.^[^ ^79^ ^]^ Copyright 2022, Wiley. D) A multiplexed chip device featured with co‐localized 3D blood vessels and proximal tubules embedded within gelatin–fibrin matrix at a spatial proximity within 100 µm. Reproduced (Adapted) under terms of the CC‐BY license.^[^ ^80^ ^]^ Copyright 2023, Royal Society of Chemistry. E) Schematic illustration of processing steps used to create 3D OPTECs‐on‐chip models, including isolating OPTECs from kidney organoids by magnetic‐activated cell sorting, expanding OPTECs, and seeding OPTECs in channels embedded within gelatin‐fibrin gel within multiplexed chip device. Reproduced (Adapted) under terms of the CC‐BY license.^[^ ^84^ ^]^ Copyright 2022, Springer Nature. F) Immunofluorescence staining of 3D kidney tissue containing proximal tubular fabricated via extrusion‐based 3D printing. Reproduced (Adapted) under terms of the CC‐BY license.^[^ ^86^ ^]^ Copyright 2019, National Academy of Sciences. G) The platform comprises 96 microfluidic devices and incorporates an integrated TEER system. Cross‐sectional view of the platform illustrates the four‐point TEER measurement setup, where stainless steel pump tubing simultaneously functions as electrodes. Reproduced (Adapted) under terms of the CC‐BY license.^[^ ^87^ ^]^ Copyright 2022, Springer Nature. H) Kidney organoids are seeded onto ECM within a perfusable chip and exposed to controlled shear stress, exhibiting enhanced vascularization during nephrogenesis. Reproduced with permission.^[^ ^88^ ^]^ Copyright 2019, Springer Nature.

Early attempts to model a renal tubule within a microfluidic device enabled continuous feeding of Madin Darby Canine Kidney cells and controlled waste removal.^[^ ^67^ ^]^ This controlled fluidic environment is valuable for providing the necessary biomimetic environments for these cells. In later studies, a porous membrane was integrated into (Ø = 0.4 µm)^[^ ^68^ ^]^ a microfluidic system while culturing human renal tubule cells at the interface of a semi‐permeable membrane sandwiched between perfusable microchannels fabricated using PDMS^[^ ^69^, ^70^, ^71^ ^]^ or etched onto glass substrates.^[^ ^72^ ^]^ These semi‐permeable membranes provided physical support to the cell barrier and enabled the simulation of its barrier function while the flow rate equivalent to 1 dyn cm^−2^ shear stress at the apical surface promoted the level of differentiation of polarized epithelial cells. The cell barriers allow transport of solutes and water, essential for reabsorption and secretion. Permeability assays in renal tubule in vitro models focus on assessing the transport of molecules and evaluating barrier functions. The enhanced maturation of living human kidney epithelial cells, including cilia forming and elongation, was observed when exposed to fluidic flow in a microfluidic device, and this enhanced maturation improved nephrotoxicity testing.^[^ ^73^, ^74^ ^]^ Microvasculature supports tubular reabsorption by enabling efficient exchange of solutes, water, and oxygen between the tubules and surrounding capillaries in vivo. Further advancements in these models incorporated vascular components. The co‐culture of PTECs and endothelial cells on a membrane within microfluidic channels allowed for the establishment of more mature models that recapitulated both cell barrier functions and intercellular crosstalk (**Figure** **4**A), as shown in a recent study.^[^ ^75^ ^]^

Currently, there have been efforts to utilize ECM as scaffolds within microfluidic chips to create more biomimetic models. A 3D‐microfluidic platform (Nephroscreen) with two lanes coated with collagen I for seeding PTECs and a lane filled with collagen I for studying transport parameters and physiology of the epithelial barrier has been developed (Figure 4B).^[^ ^76^, ^77^, ^78^ ^]^ The permeability of the barrier, assessed using fluorescently labeled dextran, showed that by day 6 following cell seeding, the tubules had established a tight barrier. In the meantime, some other microfluidic chips with multiple parallel channels can be quite suitable for building vascularized proximal tubule models. For example, Wan et al. developed a microfluidic device with multi‐compartmental channels which has been used to establish various vascularized models (Figure 4C) and has the potential for creating vascularized kidney models.^[^ ^79^ ^]^ In another study, by using templates to create parallel channels in microfluidic chips, researchers created a perfusable 3D vascularized proximal tubule‐on‐a‐multiplexed chip composed of epithelium and endothelium conduits (Figure 4D).^[^ ^80^ ^]^

However, these models still have room for improvement in terms of cell sources, complex structures, and barrier function measurement. The functionality of proximal tubule‐on‐a‐chip is particularly affected by the cell source. Currently, the majority of the PTECs are one of the following: primary human renal proximal tubule epithelial cell (RPTEC); hTERT‐immortalized human proximal tubule epithelial cell (RPTEC/TERT1); the conditionally immortalized proximal tubule epithelial cell (ciPTEC).^[^ ^81^, ^82^ ^]^ A drawback of the aforementioned sources is that they lack or do not sufficiently express many essential transporters. Transporters play a crucial role in mediating the reabsorption and secretion of substances, thereby regulating water, electrolyte, and metabolic balance. Organic cation transporter 2 (OCT2) and organic anion transporters 1 and 3 (OAT1/3) mediate the transport of drug metabolites, including organic cations and anions, across the basolateral and apical membranes of PTECs.^[^ ^83^ ^]^ Stem cell‐derived and organoid‐derived PTEC‐like cells which isolated from kidney organoids could potentially be better options. More recently, a perfusable 3D proximal tubule model composed of organoid‐derived proximal tubule epithelial cells (OPTECs) has been created (Figure 4E).^[^ ^84^ ^]^ This model provides improved drug uptake when compared to the models based on immortalized PTECs due to significantly higher expression of OCT2 and OAT1/3. This enhanced expression could be attributed to the use of OPTECs. Homan et al. fabricated a perfusable proximal tubule model through 3D extrusion bioprinting, which allows for the achievement of a biomimetic convoluted proximal tubule (Figure 4F).^[^ ^85^ ^]^ Further, Lin et al. developed a vascularized proximal tubule model through bioprinting.^[^ ^86^ ^]^ This proximal tubule model exhibited selective reabsorption, as evidenced by the progressive increase in albumin uptake over time, while the concentration of inulin‐FITC remained unchanged, suggesting the establishment of proximal tubule epithelial barrier functions. To further enhance the physiological relevance of this model, incorporating a dynamic hydrostatic gradient between the proximal tubules and peritubular capillaries in the future would better mimic the natural processes of reabsorption and secretion. Furthermore, these channels are currently much larger than their in vivo counterparts, which could also be improved in the future. OoCs can also incorporate novel sensing techniques to further enhance their application potential. For example, a high‐throughput proximal tubule platform that includes a transepithelial/transendothelial electrical resistance (TEER) measurement system has been manufactured to investigate drug nephrotoxicity (Figure 4G).^[^ ^87^ ^]^

#### 2.2.3 Kidney Organoid‐On‐A‐Chip Models

Kidney organoids, derived from iPSCs or embryonic stem cells (ESCs), are also promising in vitro tools for kidney disease research. These organoids, containing glomerular‐ and tubular‐like structures, have been combined with genetic editing techniques to study various kidney disorders. However, kidney organoids are mostly immature and avascular cultured in static.^[^ ^89^ ^]^ Culturing kidney organoids in microfluidic devices has shown promise in enhancing the expansion of the endogenous pool of endothelial progenitor cells within these organoids and promoting the formation of vascular networks with perfusable lumens within kidney organoids (Figure 4H).^[^ ^88^ ^]^ Based on this kidney organoid‐on‐a‐chip, Kroll et al. further incorporated immune cells to study cancer immunotherapy.^[^ ^90^ ^]^ The kidney organoid‐on‐chip platform paves the way for studying complex cell‐cell interactions at the organ levels.

### 2.3 Kidney‐On‐A‐Chip Models To Recapitulate Chronic Kidney Disease

As kidney damage induced by nephrotoxic substances may progress to CKD,^[^ ^91^ ^]^ many kidney‐on‐a‐chip models have been used for nephrotoxicity drug screening to facilitate the development of safer pharmaceuticals and prevent kidney damage.^[^ ^44^ ^]^ However, to our knowledge, there is a lack of kidney‐on‐a‐chip models that replicate pathological features of CKD and address the potential treatments and interventions in vitro. Multiple aetiologies, involvement of multiple cells and systems, and complex pathogenic mechanisms make it difficult to develop a physiologically relevant platform. Meanwhile, CKD involves long‐term progressive damage and remodeling, which requires sustained culture conditions that can maintain cellular homeostasis. Fortunately, more and more explorations about pathogenesis and cell phenotypes of CKD have been conducted using 2D cell cultures and animal models. In the progression of CKD, the proximal tubule, glomerulus, and interstitium display cell dysfunction and attrition.^[^ ^92^ ^]^ Proximal tubule cells exhibit dysfunction marked by impaired transport, heightened oxidative stress, and mitochondrial damage.^[^ ^93^ ^]^ Glomerular cells, including podocytes and endothelial cells, show increased permeability and cytoskeletal rearrangements, contributing to proteinuria.^[^ ^94^ ^]^ Interstitial fibroblasts become activated, driving excessive extracellular matrix deposition and fibrosis.^[^ ^95^ ^]^ These dysfunctions are driven by specific pathways, including TGF‐β signaling in fibrosis, NF‐κB in inflammation, and p53 in cell senescence.^[^ ^96^ ^]^ These features and processes could potentially be recreated in kidney‐on‐a‐chip models in the future to establish CKD models and gain further insights into human kidney disease progression. Current kidney‐on‐a‐chip models are advantageous in their ability to replicate specific segments of the nephron, particularly the structure and function of the proximal tubule or glomerular filtration barrier. For example, proximal tubule‐on‐a‐chip platforms commonly incorporate PTECs, sometimes co‐cultured with endothelial cells, to model solute transport and nephrotoxicity. Glomerulus‐on‐a‐chip models often include podocytes and glomerular endothelial cells to simulate the filtration interface. However, replicating the full structural and cellular complexity and function of an entire nephron remains technically challenging. In particular, the integration of interstitial components and crosstalk between multiple nephron segments is still limited, which constrains their application in studying multifactorial pathologies such as fibrosis in CKD. The kidney‐on‐a‐chip should expand the cell types, like fibroblasts and immune cells, for better investigating specific pathological processes of CKD. These cells can be derived from immortalized human cell lines, iPSCs‐derived kidney cells, or primary human kidney cells, with the latter two offering the advantage of capturing patient‐specific phenotypes.^[^ ^97^, ^98^, ^99^ ^]^ However, primary human kidney cells can be difficult to obtain and can tend to de‐differentiate in culture, limiting their long‐term utility for functional studies.^[^ ^100^ ^]^ Kidney‐on‐a‐chip platforms also offer significant advantages in controlling stimulus inputs, testing metabolic indicators, assessing filtration barriers, and studying cellular crosstalk. For CKD‐specific models, these hallmarks should be extended to include progressive nephron loss, fibrosis, uremic toxin accumulation, and immune cell infiltration,^[^ ^101^ ^]^ ensuring the platform captures the chronic and inflammatory nature of the disease. It is known that uremic toxins accumulate in CKD due to impaired renal clearance, which exacerbates kidney damage by inducing oxidative stress, inflammation, and fibrosis in renal cells. For example, indoxyl sulfate activates the aryl hydrocarbon receptor pathway,^[^ ^97^ ^]^ leading to tubular cell injury, while p‐cresyl sulfate contributes to endothelial dysfunction and proteinuria.^[^ ^98^ ^]^ Exposing kidney‐on‐a‐chip to these uremic toxins would be an effective strategy to study these processes in vitro.^[^ ^99^ ^]^ Perfusing systems in kidney‐on‐a‐chip also hold the great potential to realize gradient stimulation of uremic toxins, mimicking the sustained exposure seen in CKD patients. Last but not least, incorporating sensors to allow real‐time monitoring of cellular responses, such as matrix deposition and inflammatory factor levels, would also be an important future direction.

In summary, CKD models should consider including biomimetic cellular diversity, extracellular matrix dynamics, mechanical environments (e.g., shear stress), and immune cells for inflammation studies. To mimic clinical profiles more effectively, CKD models could integrate patient‐derived cells and chronic uremic toxin exposure. Through these developments, future advanced microfluidic platforms will make it possible to develop CKD models together with comorbidities for investigating complicated disease management techniques.

## 3 Joint Microphysiological Systems and Arthritis

### 3.1 Joint Microphysiological Systems

Joints are complex structures that facilitate movement and provide mechanical support in the body. Synovial joints are the most prevalent type of joint in the human body. Synovial joints consist of articular cartilage, which covers the ends of bones; the synovial membrane, which lines the joint capsule and produces synovial fluid; and the synovial fluid, which lubricates and nourishes the joint.^[^ ^102^ ^]^ Additionally, ligaments and tendons surround the joint, offering stability and enabling motion. Due to their high mobility and mechanical load, synovial joints are particularly susceptible to injury and inflammation, making them a central focus in studies of joint‐related diseases such as arthritis. Key cellular players in the joint include chondrocytes and synovial fibroblasts.^[^ ^103^ ^]^ Chondrocytes, the sole cell type in articular cartilage, are responsible for synthesizing and maintaining the ECM, which makes up over 95% of the tissue's volume and is vital for cartilage integrity and function.^[^ ^104^ ^]^ The ECM comprises collagen fibers, proteoglycans, and other molecules that together provide tensile strength and resistance to compression. Synovial fibroblasts, located in the synovial membrane, produce key components of synovial fluid, such as hyaluronic acid and lubricin, essential for joint lubrication and function.^[^ ^105^ ^]^ In disease states, these fibroblasts can become activated, contributing to inflammation and joint damage.^[^ ^106^ ^]^ Joint metabolism encompasses the dynamic processes of matrix turnover and synovial fluid production, which are critical for joint homeostasis.^[^ ^107^ ^]^ Disruptions in these metabolic activities can lead to pathologies like osteoarthritis, where cartilage degradation exceeds synthesis. Given the intricate interplay of cellular, biochemical, and mechanical factors in joint health and disease, there is a pressing need for advanced in vitro models that can accurately replicate these dynamics. We summarized current joint MPS (joint‐on‐a‐chip models) in **Table** **2** and discuss them in more detail below.

**Table 2:** Summary of joint‐on‐a‐chip models.

Model	Cell types	Fabrication	Matrix/Scaffold	Advantages	Limitations	Refs.
Strain‐controlled cartilage‐on‐a‐chip	Human chondrocytes	Soft‐lithography from PDMS	Agarose	Chip with deformable membrane and pneumatic chambers to precisely mimic mechanical loading; tunable strain magnitude and direction	Only cartilage, lack of immune components	[110]
Dual‐flow osteochondral tissue chip	Mesenchymal progenitor cells derived from iPSCs	Bilayer PDMS bioreactor with independent flow	Methacrylated gelatin	Recapitulates bone–cartilage crosstalk	No synovial component, lack of immune components	[113]
Modular synovium–cartilage chip	Fibroblasts‐like synoviocytes, human primary chondrocytes	Independent synovium and cartilage OoC units	Fibrin, Matrigel	Modules optimized separately, paracrine signaling study	Lack of mechanical stimuli and immune components	[115]
Immune‐vascularized synovium–cartilage chip	Human primary synovial fibroblasts, human primary chondrocytes, human umbilical vein endothelial cells, human primary monocytes	Multichannels	Fibrin	Model of immune cell trafficking, quantifies adhesion	Lack of mechanical stimuli	[134]

**Figure 5:**
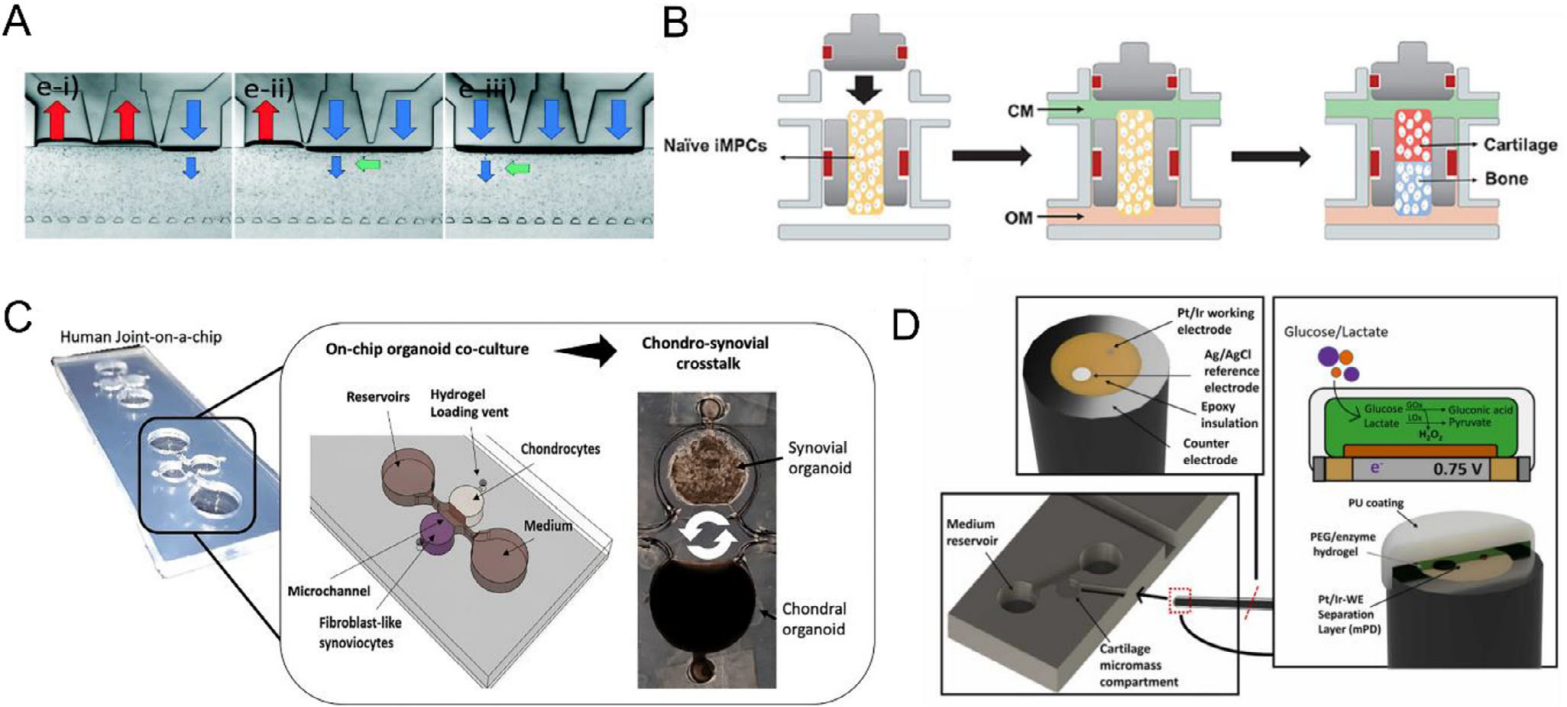
Advances of microfluidic joint‐on‐a‐chip models. A) Microscopic picture of a mechanical actuation section of the microfluidic, displaying the generated multi‐directional mechanical stimulation (shear strain‐green arrow, and compression‐blue arrow). Reproduced (Adapted) under the terms of the CC‐BY license.^[^ ^110^ ^]^ Copyright 2022, Royal Society of Chemistry. B) Chondrogenic and osteogenic media are perfused through the top and bottom flow to introduce osteochondral tissue in bioreactor. Reproduced (Adapted) under the terms of the CC‐BY license.^[^ ^113^ ^]^ Copyright 2019, Frontiers Media S.A. C) Overview of the modular joint‐on‐a‐chip coculture system composed of chondral and synovial compartments for study for inflammatory cross‐talk. Reproduced (Adapted) under the terms of the CC‐BY license.^[^ ^115^ ^]^ Copyright 2021, Royal Society of Chemistry. D) Cartilage‐on‐a‐chip platform with integrated biosensor for measuring glucose and lactate concentrations. Reproduced (Adapted) under the terms of the CC‐BY license.^[^ ^116^ ^]^ Copyright 2024, Elsevier.

Joint‐on‐a‐chip is a dynamic system designed for joint research. PDMS, a commonly used elastomeric material, is used for most joint‐on‐a‐chip devices owing to its optical transparency, gas permeability, deformability, and ease of fabrication.^[^ ^108^ ^]^ These microfluidic devices leverage emerging OoC technology to recapitulate a multifaceted joint tissue structure and microenvironment. Modeling joints involves mimicking joint microenvironments through incorporating multiple cell types (e.g., articular chondrocytes, synovial fibroblasts, monocytes, and endothelial cells), biochemical factors (e.g., synovial fluid, chemokines, and cytokines) and mechanical forces (e.g., compressive, bulk shear and hemodynamic shear stress). These components are essential for joint modeling as they collectively recapitulate the cellular, biochemical, and mechanical microenvironment of the joint. The inclusion of specific cell types allows for the study of their interactions and responses to stimuli, while biochemical factors and mechanical forces mimic the in vivo conditions that influence joint health and disease. To date, cartilage and synovium have been the primary focus of modeling efforts. Mechanotransduction regulates cartilage cellular function and responses via chondrocyte mechanoreceptors including primary cilia, ion channels, and integrins.^[^ ^109^ ^]^ Therefore, integrating strain‐controlled compression functionality into joint‐on‐a‐chip is crucial for advancing their physiological relevance. Studies have successfully developed a cartilage‐on‐a‐chip model that enables the application of strain‐controlled compression to 3D articular cartilage microtissue. To further investigate how different types of mechanical stimulation can affect a chondrocyte's phenotype and ECM production, studies have characterized the response of human chondrocytes to multi‐directional mechanical stimulation. This has been achieved via a cartilage‐on‐a‐chip system capable of applying controlled compressive and multi‐directional mechanical stimulation to 3D chondrocyte‐laden hydrogels. The regulated mechanical stimulation is achieved by using a deformable membrane connected to three individually addressed actuation chambers (**Figure** **5**A).^[^ ^110^ ^]^

Further, researchers attempted to replicate the osteochondral interface with the two tissue types cocultured for the modeling of important crosstalk between them.^[^ ^111^, ^112^ ^]^ Lin et al. successfully developed a dual‐flow bioreactor platform containing biphasic osteochondral tissues (Figure 5B).^[^ ^113^ ^]^ To enable the study of intricate interactions between tissues, a modular approach has been proposed. In this approach, each tissue in the joint is first modeled as an individual OoC device which is subsequently connected to yield the joint‐on‐a‐chip model.^[^ ^114^ ^]^ Based on this modular approach, researchers have successfully established a chip‐based three‐dimensional tissue co‐culture model that simulates the reciprocal cross‐talk between individual synovial and chondral organoids (Figure 5C).^[^ ^115^ ^]^

Recent breakthroughs integrated glucose and lactate biosensors in a human cartilage‐on‐a‐chip system, enabling real‐time detection of disease‐related metabolic shifts in chondrocytes during inflammation. This demonstrates the feasibility of in situ metabolic sensors for the study of disease progression (Figure 5D).^[^ ^116^ ^]^

### 3.2 Joint‐On‐A‐Chip Models to Recapitulate Osteoarthritis

Arthritis is the most common joint disease and remains challenging to cure due to its systemic and multi‐factorial nature.^[^ ^117^, ^118^ ^]^ As a leading cause of pain and disability, arthritis significantly impacts the quality of life of millions of people worldwide by causing joint inflammation, stiffness, and reduced mobility, requiring long‐term medical management and intervention. OA, a degenerative whole‐joint disease, is the most common form of arthritis. Aging and obesity are the main reasons for its rising prevalence. The hallmark pathological features of OA include progressive loss of articular cartilage, subchondral bone remodeling characterized by sclerosis, osteophyte formation, and in some cases, subchondral cyst development. The microenvironment of OA is altered with the increased senescence‐associated secretory phenotypes including pro‐inflammatory mediators (IL‐1β, IL‐6, IL‐7, IL‐8, IL‐18, TNF‐α) and MMPs.^[^ ^119^ ^]^ To date, several clinical techniques are available for treating cartilage injuries, including microfracture surgery,^[^ ^120^, ^121^ ^]^ osteochondral transplantation technology,^[^ ^122^ ^]^ autologous chondrocyte transplantation technology,^[^ ^123^ ^]^ stem cell therapy,^[^ ^124^, ^125^, ^126^ ^]^ and matrix‐induced chondrogenesis technology.^[^ ^127^ ^]^ However, current therapies are still limited to palliative treatments or surgical interventions.^[^ ^128^, ^129^ ^]^ Progress in developing new treatments is limited by the absence of relevant preclinical models of arthritis with clinical presentation and pathophysiological relevance to the human condition. Conventional cultures are limited to adequately capture the development and process of OA, including complex pathology, dynamics, and immune cellular responses. Therefore, joint‐on‐a‐chip has been explored to build OA models that more precisely control the pathophysiological microenvironments.

**Figure 6:**
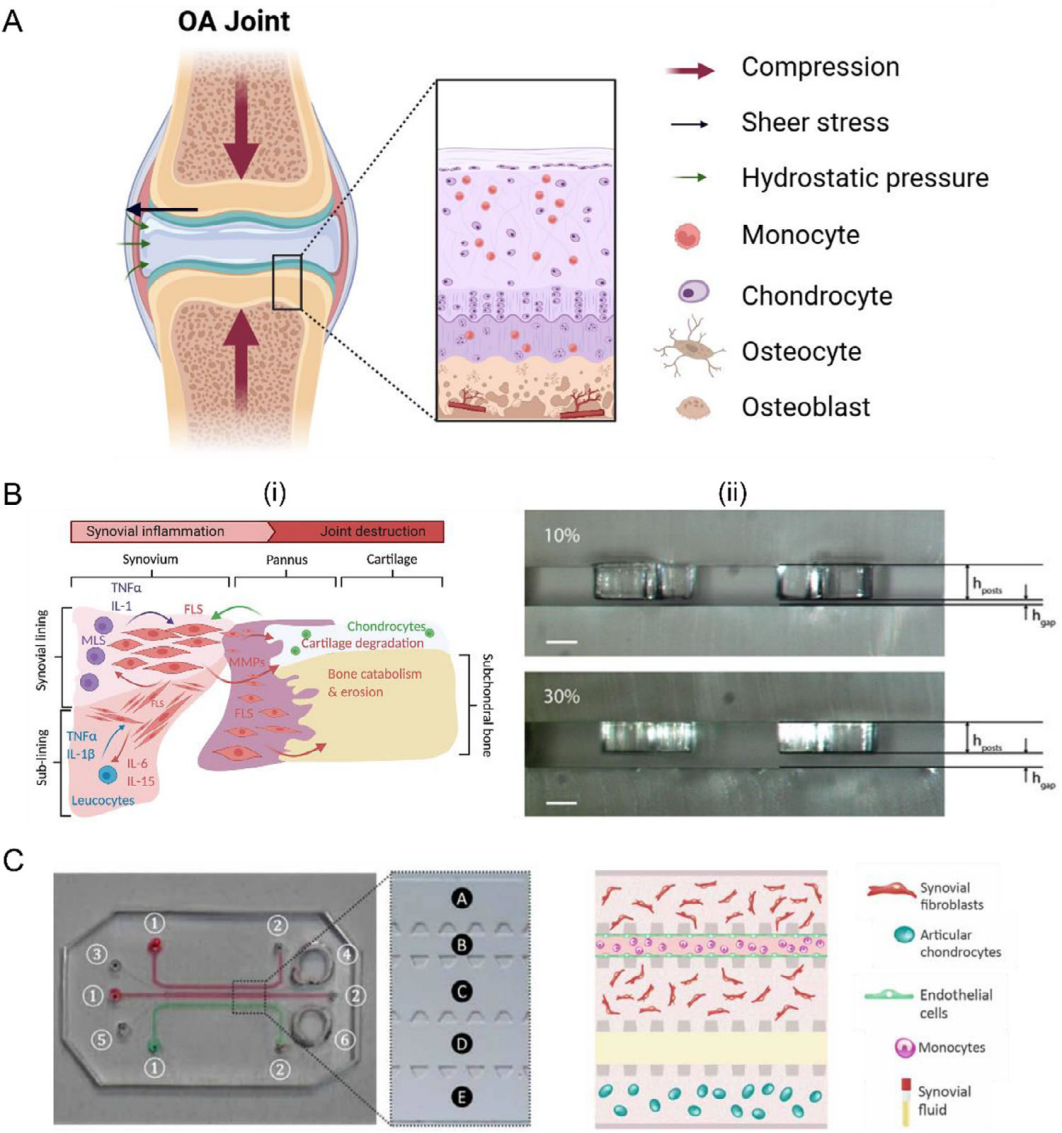
Recapitulating OA in joint‐on‐a‐chip models. A) Microenvironmental features of OA, including mechanical stimulation, multi‐tissue crosstalk, and inflammation. Figure created with BioRender.com. B) (i) Schematic illustration of the synovial inflammation leading to joint destruction; (ii) the stereomicroscope sections of the device with 10% and 30% gaps to induce OA. Reproduced (Adapted) under the terms of the CC‐BY license.^[^ ^115^ ^]^ Copyright 2021, Royal Society of Chemistry. Reproduced with permission.^[^ ^133^ ^]^ Copyright 2019, Springer Nature. C) The physical drawing and schematic illustration of OA model on joint‐on‐a‐chip with the synovium, cartilage compartments, and immune cells. Reproduced (Adapted) under the terms of the CC‐BY license.^[^ ^134^ ^]^ Copyright 2021, IOP Publishing Ltd.

The promise of joint‐on‐a‐chip lies in the ability to model key OA components (**Figure** **6**A). To model the pathology and the diversity of cells that are involved in the process, human tissues are the main source of cells in OA models. For example, Banh et al. applied mechanical compression on human OA cartilage tissues from patients using microfluidic chips, which enabled the development of synovial joint‐on‐chip models, characterized by cartilage matrix degradation.^[^ ^130^ ^]^ iPSCs and their derived organoids are emerging as promising cell sources. Rothbauer et al. established the chondro‐synovial organoid‐on‐a‐chip model.^[^ ^115^ ^]^ The cartilage exhibited enhanced architectural fidelity, and an improved differential cytokine response compared to their respective cultures, which suggests co‐culture can improve tissue physiology in arthritic disease models. Further, incorporation of biophysical and topographic cues would possibly enhance the functionality and maturity of these organoids.

To induce OA pathology, external factors, such as inflammatory cytokines^[^ ^131^ ^]^ and hyperphysiological compression,^[^ ^132^ ^]^ were applied to joint‐on‐a‐chip models (Figure 6B). For example, Lin et al. built an OA model by adding IL‐1β to the cartilage component within the microfluidic chip containing osteochondral tissue.^[^ ^113^ ^]^ The bone‐like tissue promoted the degradation of cartilage‐like tissue indicating the successful establishment of the OA model. In addition, applying 30% confined compression has been found to provide the pathology‐relevant mechanical environments involved in OA pathogenesis. This mechanical factor led to increased expression of catabolism, inflammation, and hypertrophy similar to those seen in clinical osteoarthritic tissue.^[^ ^133^ ^]^ PDMS is the main material used to fabricate the mechanical stimulation modules based on pneumatic deflection for joint‐on‐a‐chip. However, pneumatic modules rely on tubes and pumps that complicate the chip system, and PDMS readily adsorbs small molecules, limiting precise drug testing. This drawback would need to be improved for these models.

Integrating the immune environment in joint‐on‐a‐chip models is another important consideration as the synovium in OA shows an abnormal accumulation of macrophages originating from extravasated monocytes. Attempts have been made to inject monocytes into the channel of a vascularized synovium and articular cartilage joint‐on‐a‐chip model. This enabled the investigation of monocyte extravasation in OA joints (Figure 6C).^[^ ^134^ ^]^

Looking ahead, further improvements in these models may involve the modification of hydrogel scaffolds. ECM‐derived proteins have limited mechanical strength, while polymers that have outstanding mechanical properties are not able to mimic native ECM.^[^ ^133^ ^]^ Recent advances in modifying polymers with peptides or proteins have made the materials suitable for cultivating cartilage cells.^[^ ^135^ ^]^ Another strategy is to design materials that can stimulate cells to secrete instructive ECM. Integrating ECM‐triggering ligands into materials and modulating physical properties to enrich for particular components are the effective approaches for harnessing the secreted ECM.^[^ ^136^ ^]^ Additionally, accurate in situ data acquisition is critical for controlling the stimulation and producing trackable readouts. Advanced fabrication methods and devices that integrate stimulation elements and data acquisition components would contribute to models that are suitable for higher throughput and data‐intense applications like drug screening.

## 4 Multi‐Organ‐On‐A‐Chip Systems

### 4.1 Multi‐Organ‐On‐A‐Chip Systems for Improved Physiological Relevance

**Figure 7:**
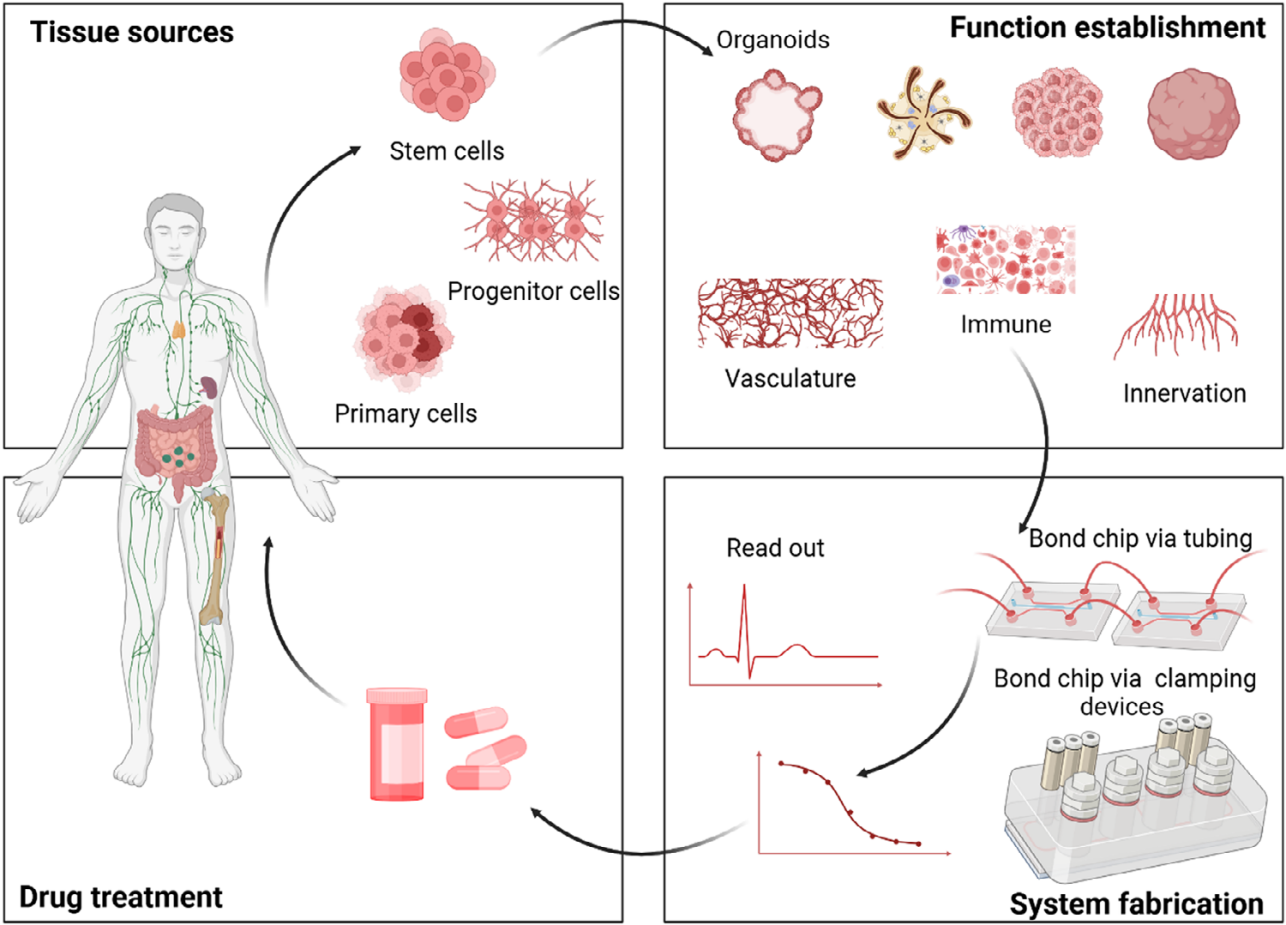
Overview of multi‐organ‐on‐a‐chip development and workflow. Figure created with BioRender.com.

Multi‐organ‐on‐a‐chip (MOoC) systems are advanced microfluidic platforms that integrate multiple organ models to simulate physiological functions and inter‐organ interactions (**Figure** **7**). They provide enhanced physiological relevance by replicating systemic interactions, enabling more accurate studies of drug efficacy, toxicity, and disease progression. The emerging modular and flexible design accommodates various organ combinations for tailored studies, making MOoC ideal for modeling complex diseases and systemic conditions. For example, in a kidney‐liver MOoC system, the hepatocyte‐specific metabolism can exacerbate the proximal tubular injury.^[^ ^137^ ^]^ Such interaction is critical for studying drug‐induced kidney injury and systemic toxin clearance, key areas of focus in kidney research. MOoC systems also pave the way for closed‐loop therapeutic approaches, where patient‐specific insights inform tailored treatment strategies. This paradigm shift underscores the transformative role of MOoC systems in advancing precision medicine.

One of the key challenges in integrating the multiple organ models is to establish the vascular barrier that connects these organ modules. To achieve vascularization across MOoC systems, Kacey et al. designed a platform that integrates mature human heart, liver, bone, and skin tissues interconnected through a recirculating vascular flow.^[^ ^138^ ^]^ This vascular integration enhances the performance of multi‐organ models by facilitating the exchange of nutrients, oxygen, and signaling molecules and promoting the organ–organ crosstalk via vascular flow. At the same time, the vascular unit helps preserve the specificity of individual organ environments. MOoC systems could also facilitate the study of how systemic circulation affects individual organs. For example, factors derived from other organs, such as natriuretic peptides derived from heart, may modulate renal filtration.^[^ ^139^ ^]^ The vascular integration in organ‐on‐a‐chip systems offers opportunities to study these interactions.

Integrating innervation is also essential for accurately modeling complex multi‐organ interactions. Neural dysfunction is a key factor in many diseases, and evaluating drugs that target the nervous system requires physiologically relevant neural integration. However, replicating the intricate architecture and functionality of native neural networks within multi‐organ systems remains a significant challenge. Recently, Osaki et al. developed an in vitro neural tissue model capable of forming inter‐regional connections by utilizing bundles of reciprocally extended axons to link tissues.^[^ ^140^ ^]^ This approach overcomes traditional physical and design constraints, enabling long‐range neural connectivity without requiring direct spatial proximity between organ modules. The addition of neural units could improve the performance of multi‐organ models by allowing the simulation of neuro‐organ interactions, thereby increasing the model's physiological accuracy. In the future, neural integration in kidney models may enable the modeling of the autonomic nervous system's regulation of renal blood flow and electrolyte balance. This control is essential for understanding how stress or neurological conditions impact kidney performance, offering insights into systemic homeostasis.

In addition to strategies for vascularization and innervation, advancements in chip fabrication techniques also drive the integration of multiple organ systems. Traditionally, individual organ chips were connected using tubing for liquid transfer, but this method suffered from drawbacks such as complex setup, poor reproducibility, bubble formation, and liquid residue adhering to tubing walls.^[^ ^141^, ^142^ ^]^ A novel clamping device approach has been introduced to simplify integration. This technique allows chips to be cultured and matured independently before being integrated, while also preventing bubble formation during operation, significantly improving efficiency and operational flexibility.

### 4.2 Multi‐Organ‐On‐A‐Chip for Comorbidity Studies

Comorbidities are a growing challenge in modern medicine. Diseases in different organs often interact in complex and dynamic ways, making it difficult to study their mechanisms and develop effective therapies using traditional models. MOoC technology has emerged as a transformative tool to address these challenges by replicating the physiological interactions between multiple organs in a controlled microenvironment.

Comorbidities, like CKD and OA, share many risk factors, including aging, obesity, diabetes, cardiovascular disease, and inflammation.^[^ ^143^ ^]^ The crosstalk between CKD and OA goes in both directions.^[^ ^144^ ^]^ CKD‐induced inflammation, metabolic disturbances, and mineral bone disorder can accelerate OA progression, while long‐term use of NSAIDs for OA pain relief may adversely affect kidney function.^[^ ^145^ ^]^ In CKD, pro‐inflammatory cytokines like IL‐6 and TNF‐α, released due to kidney damage, circulate systemically and promote OA progression by exacerbating synovial inflammation and upregulating MMP‐13, leading to cartilage breakdown.^[^ ^146^ ^]^ Conversely, OA's synovial inflammation releases pro‐inflammatory cytokines, worsening kidney injury via glomerular inflammation and fibrosis.^[^ ^147^ ^]^ Treatment choices further complicate this interplay. For instance, NSAIDs used for OA pain relief can impair renal blood flow, exacerbating CKD. Meanwhile, the development of mineral and bone disorders, characterized by disrupting calcium‐phosphorus balance, can lead to cartilage calcification and subchondral bone changes that accelerate OA.^[^ ^148^ ^]^ The fact that CKD is significantly more prevalent in OA patients than in the general population has been recognized.^[^ ^149^, ^150^ ^]^ However, their interactions are not yet fully understood, and translating drugs of interest into clinical practices for patient treatment remains a significant challenge. MOoCs offer a controlled platform to explore these dynamics, allowing researchers to simulate drug administration sequences and assess their impact on both conditions, guiding optimized treatment strategies.^[^ ^151^ ^]^

Despite this potential, to our knowledge, few MOoCs currently include both kidney and joint tissues. Notably, successful kidney‐incorporating MOoCs with other organs (e.g., kidney‐liver, gut‐kidney) have been developed.^[^ ^152^ ^]^ Kidney‐incorporating MOoCs are focused on researching drug metabolism, excretion, and toxicity.^[^ ^153^ ^]^ MOoCs consisting of kidney and liver, the most popular combination, have been developed to investigate the hepatotoxicity and nephrotoxicity of compounds since the liver is the main organ for drug metabolism and drug metabolites are excreted through the kidneys. Lin et al. developed a MOoC with kidney and liver to investigate expressions of epithelial markers, cilia, and transporter localization after adding cyclosporine A.^[^ ^154^ ^]^ This study demonstrated MOoCs provide a platform for assessing the nephrotoxicity of drugs and their metabolites. A gut‐kidney axis‐on‐chip model was used to study microbiome‐driven kidney damage and therapeutic strategies targeting the gut as the gut microbiome significantly influences kidney health by producing uremic toxins that exacerbate CKD.^[^ ^155^ ^]^ Maschmeyer et al. developed a MOoC system consisting of 4 organs, including the kidney, liver, intestine, and skin in connected microfluidic channels.^[^ ^156^ ^]^ The levels of glucose homeostasis in the organs were surprisingly stable during the 28 days co‐culture. These advanced studies suggest that MOoCs promote mimicry of pharmacokinetic profiles and cellular responses.

Various microfluidic strategies discussed in earlier sections can potentially be used to build CKD‐OA MOoCs for monitoring key cellular or organ‐level metrics of interest. Aging, a critical chronic disease driver, induces cellular senescence in organs, reducing repair capacity and exacerbating diseases. CKD‐OA MOoCs can include cells from older donors or induce senescence to mimic the aging patient population where CKD and OA prevalence peaks. Hydrogel materials, such as Collagen type I and II, fibrin, decellularised extracellular matrix, could potentially be used to mimic the extracellular matrix of kidney tissues. Polymers with surface modifications and excellent mechanical properties could be better suited for mimicking the ECM of load‐bearing tissues like joints. The kidney and joint can be co‐cultured in separate microfluidic chambers, allowing preservation of their individual microenvironments while enabling systemic interaction through interconnected microchannels.

Generally, the disease phenotypes in CKD include renal fibrosis, cellular senescence, disruptions in mineral metabolism, and alterations in kidney function. The main disease phenotypes in OA include cellular senescence, abnormal secretion of MMPs, increased pro‐inflammatory cytokines secretion, and emergence of chondrocyte clusters.^[^ ^157^ ^]^ To guide future CKD‐OA MOoC development, measurable and quantitative criteria should reflect the pathological characteristics of both diseases. Key hallmarks include inflammatory cytokines (IL‐6, TNF‐α, IL‐1β), kidney function markers (albuminuria, GFR), OA progression markers (MMP‐13), and fibrosis (collagen I deposition). Incorporating advanced real‐time monitoring techniques into the MOoC will improve the efficiency and accuracy of monitoring cellular morphology and biochemical factors. Techniques like biosensor integration for real‐time cytokine monitoring and microscopy for morphological analysis will provide quantitative insights. Raman micro‐spectroscopy may be integrated as a non‐invasive method to monitor real‐time biochemical changes for the in vitro models.^[^ ^158^ ^]^ Advanced AI‐empowered algorithms will potentially further improve the interpretation of the readouts.^[^ ^159^, ^160^ ^]^ In the future, realizing high throughput and sensing integration simultaneously would enable us to effectively explore the underlying causes of comorbid CKD and OA. Besides, microchannels could be implemented to mimic the circulatory system, allowing soluble factors (e.g., cytokines, uremic toxins) to diffuse between kidney and joint compartments, facilitating the exploration of bidirectional crosstalk. For instance, researchers can use microchannels to replicate how CKD‐induced IL‐6 affects joint cartilage or how OA‐driven TNF‐α impacts kidney fibrosis, shifting focus from isolated disease hallmarks to integrated systemic interactions. MOoCs enable precise spatiotemporal control of drug administration, allowing researchers to selectively introduce therapeutic agents into one compartment and monitor downstream effects in interconnected compartments. This compartmentalized approach provides a powerful platform to investigate kidney‐joint interactions under pharmacological intervention, offering critical insights into systemic side effects and informing the development of safer, more targeted treatment strategies for comorbidities.

## 5 Conclusion

In this review, we summarize recent advances in OoC models for kidneys and joints, discuss the modeling strategy for CKD and OA, and explore the challenges and opportunities of OoCs in comorbidity research. The prevalence of CKD, OA, and other comorbidities is increasing as the world's population ages. However, models for the study of these comorbidities have been rare. The development of advanced OoC models would be promising for future studies on comorbidities. We comprehensively discussed the available tools for creating kidney‐on‐a‐chip platforms. Notably, while various kidney models have been developed, in vitro CKD models are still underdeveloped. The highly flexible and tunable kidney‐on‐a‐chip platforms hold the potential to incorporate the key cells involved and precise environmental controls to recapitulate the pathological features, mechanical and biochemical microenvironment of CKD. The modeling of OA in joint‐on‐a‐chip systems has made great progress. Our systematic discussion of microfluidics, cell sources, biofabrication technologies, and modeling methods for CKD and OA may provide valuable guidance for developing comorbidity models. Meanwhile, we discussed the progress of MOoC platform development and their implications for building integrated comorbidity models and for advancing personalized treatment strategies. We believe the further development of MOoCs will help us gain pathological insights into comorbidity interactions and facilitating the development of safer and more efficient therapeutics.

## Conflict of Interest

The authors declare no conflict of interest.
